# MicroRNA-150 (miR-150) and Diabetic Retinopathy: Is miR-150 Only a Biomarker or Does It Contribute to Disease Progression?

**DOI:** 10.3390/ijms232012099

**Published:** 2022-10-11

**Authors:** Gladys Y.-P. Ko, Fei Yu, Kayla J. Bayless, Michael L. Ko

**Affiliations:** 1 Department of Veterinary Integrative Biosciences, College of Veterinary Medicine and Biomedical Sciences, Texas A&M University, College Station, TX 77843, USA; 2 Texas A&M Institute for Neuroscience, Texas A&M University, College Station, TX 77843, USA; 3 Department of Molecular & Cellular Medicine, College of Medicine, Texas A&M University, College Station, TX 77843, USA; 4 Department of Biology, Division of Natural and Physical Sciences, Blinn College, Bryan, TX 77802, USA

**Keywords:** microRNA, diabetes, oxidative stress, inflammation, apoptosis, pathological angiogenesis, retinopathy

## Abstract

Diabetic retinopathy (DR) is a chronic disease associated with diabetes mellitus and is a leading cause of visual impairment among the working population in the US. Clinically, DR has been diagnosed and treated as a vascular complication, but it adversely impacts both neural retina and retinal vasculature. Degeneration of retinal neurons and microvasculature manifests in the diabetic retina and early stages of DR. Retinal photoreceptors undergo apoptosis shortly after the onset of diabetes, which contributes to the retinal dysfunction and microvascular complications leading to vision impairment. Chronic inflammation is a hallmark of diabetes and a contributor to cell apoptosis, and retinal photoreceptors are a major source of intraocular inflammation that contributes to vascular abnormalities in diabetes. As the levels of microRNAs (miRs) are changed in the plasma and vitreous of diabetic patients, miRs have been suggested as biomarkers to determine the progression of diabetic ocular diseases, including DR. However, few miRs have been thoroughly investigated as contributors to the pathogenesis of DR. Among these miRs, miR-150 is downregulated in diabetic patients and is an endogenous suppressor of inflammation, apoptosis, and pathological angiogenesis. In this review, how miR-150 and its downstream targets contribute to diabetes-associated retinal degeneration and pathological angiogenesis in DR are discussed. Currently, there is no effective treatment to stop or reverse diabetes-caused neural and vascular degeneration in the retina. Understanding the molecular mechanism of the pathogenesis of DR may shed light for the future development of more effective treatments for DR and other diabetes-associated ocular diseases.

## 1. Overview of Diabetic Retinopathy

Diabetes is a disease characterized by hyperglycemia associated with either insulin deficiency or resistance, and the incidence of diabetes is projected to increase to 33% of the US population by 2050 owing to the obesity epidemic [1], of which 90–95% of diabetic patients will have type 2 diabetes (T2D) [2]. Diabetic retinopathy (DR) is a chronic complication associated with both T1D and T2D. It impacts 4.2 million people in the US and 93 million worldwide [3] and is a leading cause of blindness among the working population in the US [4]. Overall, DR is diagnosed in 30% of diabetic patients: approximately 90% of T1D and 60% of T2D patients develop DR [5]. The risk factors for developing DR include the duration of diabetes (≥20 years), poor control over blood glucose levels, hypertension, and obesity [6].

Clinically, DR has been diagnosed and treated as a vascular disease, but it also affects the neural retina [7]. Diabetic insults impair the integrity of retinal microvasculature and induces pathological angiogenesis [8]. Depending on the severity of the vascular pathologies, DR is divided into non-proliferative and proliferative phases clinically. Non-proliferative DR (NPDR) manifests mild-to-moderate vascular abnormalities including microaneurysms, intraretinal hemorrhages, and venous beading, while proliferative DR (PDR) displays neovascularization and pre-retinal hemorrhages [5]. In addition, the decreased retinal light responses recorded by electroretinogram (ERG) correlate with more severe vascular pathologies in NPDR patients [9]. As chronic diabetic conditions adversely impact the neural retina and retinal vasculature, the interaction between retinal neurons and vascular cells could further contribute to the pathogenesis of DR.

Laser photocoagulation is a commonly used therapy for PDR but is invasive and often induces blind spots in the retina [5]. The most used therapy for DR is the intraocular injections of anti-vascular endothelial growth factor (VEGF) agents [10]. However, nearly 30% of patients do not respond well to anti-VEGFs [11,12], and less than 50% of patients have improved vision after 1–2 years of anti-VEGF therapies [13]. As repeated anti-VEGF treatments are needed to conquer the recurrent neovascularization, they often cause unwanted side effects, including retinal detachment [13]. In addition, current therapies for DR mainly target neovascularization at the later stages of DR and rarely restore normal visual function [5]. Therefore, it is critical to understand the mechanisms underlying the pathogenesis of DR and develop therapeutic strategies to either target the early stage of DR or to prevent its development.

### 1.1. Retinal Neural Dysfunction and Degeneration in DR

Neural dysfunction and degeneration occur early in the diabetic retina. Distorted color vision in patients with early diabetes was previously reported [14,15,16], and dysfunction of the neural retina can be detected in diabetic patients by ERG before any vascular pathologies are detected [17,18,19]. Diabetic patients without DR vascular complications usually have lower ERG amplitudes and longer implicit times than healthy subjects [20]. Apoptotic non-vascular cells can be found in the retina of diabetic patients before the onset of DR [21]. In T1D patients without retinopathy, the thickness of the retinal nerve fiber layer (NFL) decreases compared with healthy subjects, suggesting the loss of axons from retinal ganglion cells (RGCs) [22], and the dampened vision correlates with thinner neural layers [23]. While most apoptotic neurons are found in the retinal ganglion cell layer (GCL), the outer nuclear layer (ONL; photoreceptors) also displays apoptosis [24]. In patients with six years of diabetes duration, the apoptotic cells increase in the retina from the ONL to GCL [25]. The apoptotic markers are detected in the retina of diabetic patients, including caspase-3, a protease for apoptosis, in the GCL and Fas ligand (FasL) in major retinal layers (from NFL to ONL) [26]. Adolescents with T2D for an average of two years have reduced retinal thickness measured by optical coherence tomography (OCT) and dampened light responses measured by ERG [27]. In T2D patients without retinopathy, the thickness of retinal layers from GCL to the outer plexiform layer (OPL) decrease after one year of follow-up [28]. In T2D patients with mild NPDR (microaneurysms), the thicknesses of retinal NFL, GCL, and inner plexiform layer (IPL) decrease, indicating neurodegeneration in the initial stage of DR [29,30]. The decreased thickness of NFL correlates with the severity of DR in T2D patients, suggesting that neurodegeneration in the diabetic retina might exacerbate the development of DR [31].

In diabetic animal models, decreased thickness of the retinal inner nuclear layer (INL) and IPL occur in the retina of T1D mice (Ins2Atita) with increased expression of caspase-3 [32]. The number of cells decreases while the expression of caspase-3 increases in the GCL after ten weeks of diabetes in streptozotocin (STZ)-induced T1D mice [33]. In a mouse model of T2D (KKAY), the terminal UTP nick-end label (TUNEL) staining shows an increased number of apoptotic neurons in the GCL [34], and neuronal apoptosis in the retina occurs in T2D (db/db) mice starting from twenty weeks old [35]. These findings reveal the degeneration of the inner retina in diabetes, which may explain the dampened light response reflected by the decreased amplitude and increased implicit time of ERG b-waves [36,37].

Among retinal neurons, photoreceptors undergo apoptosis shortly after the onset of diabetes [38,39]. The ERG a-waves from diabetic patients [40] and animals [41] display decreased amplitudes and increased implicit times compared with healthy counterparts, which indicate a diabetes-induced impairment to the photoreceptors. In T2D patients, decreased thicknesses of the ONL and the inner and outer segments of photoreceptors are associated with the development of retinopathy [28]. In patients with metabolic syndromes, the thickness of the photoreceptor layer decreases compared with healthy subjects [42]. Apoptotic photoreceptors can be detected in STZ rats 4 weeks after the onset of diabetes [38], while the thickness of the ONL decreases in STZ mice after 10 weeks of diabetes [33]. Electron microscopy shows disorganization and degeneration of the outer segments of photoreceptors in STZ rats [43]. In addition, 28-week-old T2D mice (db/db) have decreased thickness of the ONL accompanied by dampened light responses on ERG [35]. Interestingly, long-term diabetic patients with retinitis pigmentosa (RP), a genetic disease with loss of photoreceptors, rarely develop DR even though these patients develop other diabetes-related vascular diseases [44,45]. In a mouse model of RP, during the period when photoreceptors are undergoing apoptosis, the retinal vasculature is also degenerating. Once the photoreceptors are completely lost, the vascular degeneration stops [46]. Hence, photoreceptor apoptosis not only contributes to the neural dysfunction under diabetes but may also adversely impact diabetic microvascular complications [46]. However, how diabetic insults cause photoreceptor apoptosis remains unclear. 

### 1.2. Retinal Vascular Degeneration and Complications in DR

The TUNEL labelling of microvascular networks in trypsin-digested retinas from diabetic patients shows significantly increased apoptotic endothelial cells and pericytes compared with healthy subjects [47,48]. The loss of pericytes and the formation of acellular capillaries are the major signs of microvascular degeneration that occurs at an earlier stage of DR [7]. Acellular capillaries contain only the basement membrane and remnants of endothelial cells without nuclei, which are typical pathological changes found in trypsin-digested diabetic retinas [49]. Another sign of microvascular degeneration is the loss of pericytes or the existence of “ghost” pericytes that appear as light-stained pockets around the basement membrane [50]. Degenerated vessels are detected in the eyes of patients with mild NPDR and may contribute to the formation of microaneurysms [51]. 

In alloxan-induced T1D rats, the number of apoptotic vascular cells and acellular capillaries are increased in trypsin-digested retinas [52]. Apoptotic endothelial cells are also increased in the retina of STZ-T1D rats, but inhibition of FasL dampens apoptosis [53]. Apoptotic pericytes expressing the pro-apoptotic BCL2 associated X (BAX) protein, an apoptosis regulator, can be detected in the retina of diabetic patients [54]. The TUNEL-positive pericytes are detected in the retina of STZ-T1D rats [55], while a loss of pericytes is found in db/db-T2D mice [56]. The number of acellular capillaries and ghost pericytes are increased in the retina of STZ-T1D and Zucker-T2D rats along with elevated activities of caspase-3, and inhibition of tumor necrosis factor (TNF-α), a pro-inflammatory cytokine that can trigger necrosis or apoptosis, alleviates those pathological changes [57]. 

Microvascular degeneration may contribute to the breakdown of the blood–retina barrier (BRB) [58] and exacerbate the decrease of blood flow and local hypoxia in the diabetic retina [59]. Retinal hypoxia stimulates the secretion of angiogenic factors such as VEGF from various cell types in the retina, including astrocytes and Müller glia [60]. The upregulated VEGF eventually leads to pathological angiogenesis and neovascularization in DR.

## 2. Pathogenesis of Diabetes-Induced Degeneration in Retinal Neurons and Microvasculature: Oxidative Stress, Inflammation, and Glutamate Excitotoxicity

### 2.1. Oxidative Stress in the Diabetic Retina

Oxidative stress is due to overproduction or decreased removal of reactive oxygen species (ROS) in cells. Mitochondria are the major organelle that produce OS during oxidative phosphorylation [61]. Normally, the electrons donated by reduced nicotinamide adenine dinucleotide (NADH) and flavin adenine dinucleotide (FADH_2_) are transferred from complex I to complex IV in the inner mitochondrial membrane by coenzyme Q and cytochrome C. Meanwhile, protons are transferred to the intermembrane space to generate a gradient of protons between the mitochondrial matrix and the intermembrane space. The influx of protons drives the synthesis of ATP while the electrons are consumed to produce H_2_O [62]. Diabetes-associated hyperglycemic conditions promote the generation of electron donors NADH and FADH_2_, which pushes the proton gradient to the threshold and ultimately hinders the transfer of electrons. Alternatively, electrons are provided to O_2_ by coenzyme Q to generate superoxide and ROS [63]. Nicotinamide adenine dinucleotide phosphate (NADPH) also donates electrons to O_2_ through the membrane proteins NADPH oxidases (Nox) [64]. Under diabetic conditions, protein kinase C (PKC) is activated that further increases the activity of Nox [65]. The Nox proteins are highly expressed in the vasculature, so ROS generated from Nox contributes to impaired vascular function [66]. The increased ROS in mitochondria promotes the opening of mitochondrial permeability transition pores (mPTP) and increases the release of cytochrome C [67], which eventually induces apoptosis. 

Eight-hydroxydeoxyguanosine (8-OHdG) is a biomarker for oxidative stress-induced DNA damage which is increased in the vitreous of T2D patients indicating upregulated oxidative stress in the retina [68]. Antioxidant reagents have been used to alleviate the diabetes-caused apoptosis of retinal neurons and endothelial cells [61]. Glutathione (GSH) is a major endogenous antioxidant that removes ROS and suppresses oxidative stress. In STZ-induced diabetic mice, GSH is decreased in the mitochondria of the retina, which correlates to the increase of degenerated (acellular) retinal capillaries [69]. Antioxidant α-lipoic acid treatment decreases the apoptotic microvascular cells after 11 months of STZ-induced diabetes, and it decreases 8-OHdG and increases GSH in the retina [70]. Treatment with the antioxidant lutein reduces the activity of caspase-3, an enzyme leading to apoptosis, and alleviates STZ-induced neural dysfunction [71]. 

### 2.2. Inflammation in the Diabetic Retina

Inflammation is a hallmark of diabetes that manifests in the diabetic retina [72]. A functional blood–retina barrier (BRB) in the diabetic retina protects the neural retina from the invasion of immune cells in the circulation [73]. Before the breakdown of the BRB in the advanced stages of DR, the retinal glial cells, neurons, and endothelial cells are the major sources of diabetes-induced inflammation [74,75,76]. The innate immune system in the retina responds to diabetic insults by activating microglia and secreting pro-inflammatory molecules. Retinal microglia are the resident immune cells derived from monocytes. In addition, the circulating macrophages can differentiate into microglia upon stimulation by low expression of CD45. Resting retinal microglia reside in the inner and outer plexiform layers with ramified shapes, while the activated microglia change to an amoeboid shape and migrate to various retinal layers [73]. 

In mouse retinas under oxidative stress, the apoptosis of photoreceptors increases concurrently with the activation of microglia in the photoreceptor layers. 

Activated microglia secrete pro-inflammatory factors, including TNF-α and interleukins (ILs), which exacerbate inflammatory reactions and promote apoptosis in vascular cells and neurons [77]. In cultured human retinal endothelial cells (HRECs), treatments of IL-1β and TNF-α increase the activities of caspase-3 and caspase-8 [78], activate the pro-inflammatory nuclear factor kappa B (NFĸB), and upregulate the expressions of intracellular adhesion molecule (ICAM)-1 and vascular cell adhesion molecule (VCAM)-1 [79]. Increased activities of caspase-3 and -8 indicate an increase of cell apoptosis, and upregulations of ICAM-1 and VCAM-1 induce leukostasis (adherence of leukocytes) in the retinal vessels and promote further inflammatory reactions by mediating the migration of leukocytes [80]. Knocking out the type 1 interleukin-1 receptor (IL1R1) in STZ-diabetic mice largely decreases the activities of the caspases and the number of acellular capillaries [81], and inhibition of TNF-α decreases the activities of caspase-3 and -8 and the apoptosis of RECs in STZ-induced diabetic rats. Furthermore, diabetic mice with deficient TNF-α receptors (TNFR1 and TNFR2) have decreased acellular capillaries and increased pericytes compared with wild-type diabetic mice [82]. These data clearly demonstrate that inhibition of pro-inflammatory factors or their receptors will decrease apoptosis of endothelial cells and preserve the retinal microvasculature.

Increased IL-1β and TNF-α also induce apoptosis in retinal neurons. Under hypoxia, the expressions of IL-1β and TNF-α in retinal microglia and the corresponding receptors IL-1R1 and TNFR1 in retinal ganglion cells (RGCs) are increased, which leads to apoptosis of RGCs. Neutralizing IL-1β and TNF-α with antibodies suppresses the apoptosis of RGCs [83]. In the retinal degeneration 1 (rd1) mouse model, blocking the downstream signaling of interleukin-1 receptors (IL-1R) alleviates the degeneration of photoreceptors and improves the light responses of the retina [84]. Thus, diabetes-elicited secretion of pro-inflammatory molecules from microglia triggers apoptosis in the neural retina. 

The astrocytes and Müller glia cells also contribute to the inflammation in the diabetic retina. Under hyperglycemia, astrocytes have increased activation of NFĸB and production of ROS as well as elevated levels of pro-inflammatory factors including IL-1β, TNF-α, and monocyte chemoattractant protein-1 (MCP-1) [85]. The MCP-1 may further recruit and activate microglia, which accelerate the local inflammatory response [86]. The Müller glial cells in the retina of STZ-induced diabetic rats have increased expression of pro-inflammatory factors, such as ICAM-1 [87]. Increased IL-1β in the diabetic retina can stimulate the expression of IL-6 and activate NFĸB in Müller cells, suggesting that the Müller cells mediate the exacerbation of inflammation in the diabetic retina [88]. In addition, the production and secretion of anti-inflammatory pigment epithelium-derived factor (PEDF) [89] are decreased in Müller cells under hyperglycemic [90] or hypoxic [91] conditions, but the expression of pro-inflammatory VEGF in Müller cells is increased under these conditions [92,93]. Taken together, microglia, astrocytes, and Müller cells are all involved in diabetes-elicited inflammation and contribute to apoptosis in the neural retina.

### 2.3. Glutamate Toxicity in the Diabetic Retina 

One mechanism of neuronal apoptosis in the diabetic retina is glutamate excitotoxicity. Glutamate, an excitatory neurotransmitter, mediates the synaptic transmission in the retina [94]. Uptake of glutamate in the synaptic cleft by neurons and glial cells is necessary to maintain the concentration of extracellular glutamate and cease the activation of the postsynaptic receptors [95]. In the diabetic retina, glutamate [96] and its receptors are upregulated [97]. In addition, the activity of glutamate transporters is reduced in Müller glia cells under diabetes [98,99]. Increased glutamate release and reduced glutamate transporters induce extended activation of the glutamate receptors allowing excessive influx of calcium into neurons [100]. The elevated intracellular calcium is transported into the mitochondrial matrix and activates the PTP, which facilitates the release of cytochrome C and production of ROS and leads to the apoptosis of neurons [101]. Hence, diabetes-elicited changes in glutamate, its receptors, and glutamate transporters in the retina cause glutamate toxicity that also contributes to neuronal apoptosis.

## 3. MicroRNAs and DR

### 3.1. Overview of microRNAs and DR 

MicroRNAs (miRs) are short, non-coding, single-stranded RNAs approximately 23 nucleotides in size, and they target one or more downstream messenger RNAs (mRNAs) causing post-transcriptional degradation or translational repression [102,103,104]. The mature miR is derived from a precursor sequence transcribed from the genome by either RNA polymerase II or III, and miR expression shows tissue- and developmental-stage-specific patterns [102,103]. MicroRNAs inhibit the translation of target mRNAs by preventing the initiation of translation [105,106,107,108], inhibiting the elongation of translation [109,110,111], and inducing the degradation of target mRNAs [102,103,112].

MicroRNAs represent a set of modulators that regulate metabolism, inflammation, and angiogenesis [113], and they have also been linked to DR [114,115]. Changes of miR levels in various organs or blood have been reported in diabetic patients and animals, and in particular, changes in circulating or retinal miRs correlate to some disease progressions and have been suggested as biomarkers for chronic diseases associated with diabetes including DR [114,116]. In STZ-induced diabetic rats, at least 86 miRs are altered in the retina [115,117,118]. Patients with T1D have circulating miR-29a, miR-148a, miR-181a, and miR-200a upregulated, while miR-21a, miR-93, miR-126, and miR-146a are downregulated [119]. The level of miR-126 negatively correlates with the risk of developing PDR [120]. The decreased miR-150 and increased miR-30b detected from the plasma of T1D patients are associated with the development of DR [121]. In the plasma of T2D patients and obese-hyperglycemic mice (ob/ob), the levels of miR-15a, miR-20b, miR-21, miR-24, miR-126, miR-191, miR-197, miR-320, miR-486, and miR-150 are decreased [122]. The downregulation of miR-20b in the serum of T2D patients correlates with the development of DR and may be used to predict the severity of DR [123]. While diabetes-associated changes of miRs clearly demonstrate a correlation between miRs and DR progression, few miRs have been shown to directly contribute to the pathogenesis of DR. 

### 3.2. Evidence of miRs Involved in Inflammation, Oxidative Stress, and Apoptosis in DR

As described in previous sections, inflammation, oxidative stress, and cell apoptosis are part of the pathogenesis of DR, and changed miRs in the blood or retina could be involved in these processes [124]. In cultured retinal ganglion cells (RGCs) treated with a high concentration of glucose (HG), the expression of miR-495 is increased. Overexpression of miR-495 further exacerbates the HG-induced RGC apoptosis, while its inhibition protects RGCs against cell death [125]. In the retina of high-fat-diet-induced T2D rats, retinal miR-93-5p is decreased. Overexpression of miR-93-5p in the diabetic retina alleviates the microvascular degeneration, downregulates pro-inflammatory factors (IL-1β, IL-6, and TNF-α), and elevates antioxidant levels, including GSH and superoxide dismutase (SOD) [126]. MiR-21 is decreased in the retina of T2D mice (db/db) and in RECs treated with palmitic acid, a condition mimicking an extracellular high-fat environment. Knocking out miR-21 in T2D mice alleviates the degeneration and leukostasis of the retinal microvasculature, decreases the levels of pro-inflammatory factors (TNF-α and VCAM-1), and upregulates the antioxidant PPARα in the retina [127]. Overexpression of miR-145 in cultured RECs alleviates the HG-induced apoptosis in part because the activation of toll-like receptor 4 (TLR4) mediates inflammatory responses and promotes the activation of NFĸB [128], and TLR4 is a downstream target of miR-145. In HG-treated RECs, overexpression of miR-145 suppresses the expression of TLR4 and inhibits the activation of NFĸB and production of other pro-inflammatory factors (IL-1β and TNF-α) [129].

In STZ-diabetic rats, miR-195 is increased in the retinal GCL, INL, ONL, and RECs compared with non-diabetic rats. The upregulation of miR-195 also occurs in cultured RECs treated with HG. The expression of manganese superoxide dismutase (MnSOD), an endogenous antioxidant, is decreased in the STZ-diabetic retina and cultured RECs treated with HG, which correlates with increased oxidative stress and apoptosis [130,131]. Inhibition of miR-195 mitigates the STZ-diabetes- and HG-induced suppression of MnSOD, thus alleviates diabetes and HG-elicited apoptosis [132]. MiR-146a is downregulated in the circulation of T2D patients [133] and also decreased in cultured RECs treated with HG [134]. Decreased miR-146a correlates with escalated inflammation [135]. In STZ-diabetic rats, intraocular injection of miR-146a suppresses the diabetes-induced increase of the pro-inflammatory intercellular adhesion molecule 1 (ICAM1) and mitigates the damage to retinal light response and microvascular integrity [118]. Overexpression of miR-146a inhibits the inflammatory response in HG-treated RECs by suppressing the expressions of TLR4, phosphorylated NFĸB, and TNF-α and blocking the downstream signaling of TLR4 [134]. MiR-15a is decreased in the RECs of diabetic patients compared with non-diabetic subjects. Overexpression of miR-15a in the mouse retina inhibits the expression of pro-inflammatory factors, including IL-1β, IL-6, and TNF-α [136]. MiR-20b is decreased in the serum of T2D patients compared with healthy subjects, and T2DR patients have further decreases of miR-20b compared with T2D patients without retinopathy [123]. Overexpression of miR-20b-3p in the eyes of STZ-diabetic rats alleviates the visual dysfunction as well as neural and vascular degeneration in the retina by reducing BAX but increasing BCL-2, thus reducing apoptosis in the retina of STZ-diabetic rats. In addition, the expressions of pro-inflammatory factors (IL-1β and TNF-α) are downregulated by overexpressing miR-20b-3p in the diabetic retina [137]. These examples further demonstrate the crucial roles of miRs in the development of DR. (Table 1).

## 4. MicroRNA-150 and DR

### 4.1. Decreased microRNA-150 (miR-150) Is Correlated with the Development of DR

**Figure 1:**
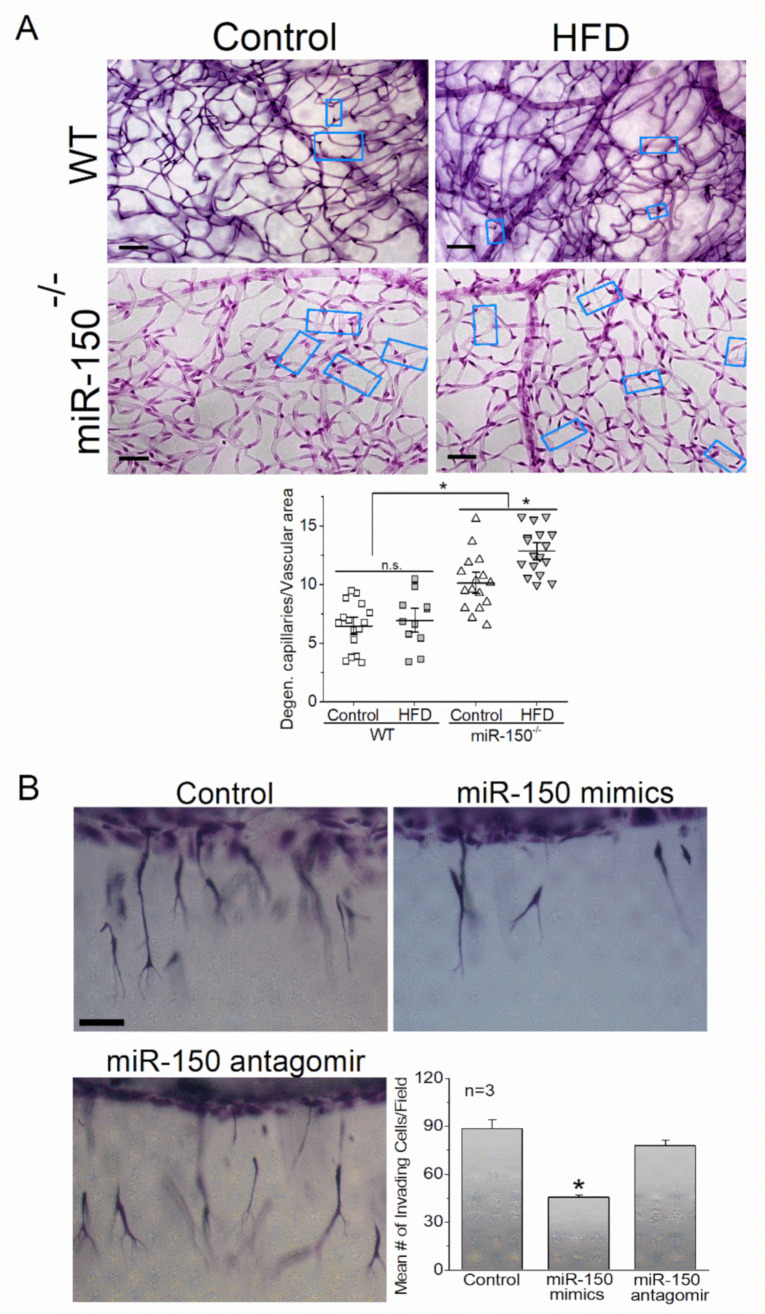
MiR-150 is anti-angiogenic. (**A**) Deletion of miR-150 exacerbates high-fat-diet (HFD)/obesity-associated DR microvascular complication. The retinas were taken from wild-type (WT) and miR-150^−/−^ that were fed with a normal (control) or an HFD (60% fat calories) for six months. The whole mount retinas were trypsin-digested and stained with H&E stain. Global knockout of miR-150 further promotes microvascular complications (such as capillary degeneration) in the vascular retina. * indicates the statistical differences from the WT control. Data taken from Yu et al., 2020 [142]. (**B**) Overexpression of miR-150 dampens endothelial sprouting, a foundation step of angiogenesis. Three-dimensional (3D) endothelial cell sprouting/invasion assays were used in this study. Primary human umbilical vein endothelial cells (HUVECs) were first transfected with 50 nM miR 150-5p mimics (miR-150 mimics), 30 nM miR 150-5p antagomir (miR-150 inhibitor), or vehicle (control) using a transfection kit. Forty-eight hours after transfections, cells were seeded onto collagen matrices containing sphingosine 1-phosphate, type I collagen isolated from rat tails, and basal fibroblast growth factor (bFGF) and VEGF. Cells were allowed to invade overnight at 37 °C with 5% CO_2_ before fixation with 3% glutaraldehyde. After 24 h of invasion, overexpression of miR-150 (miR-150 mimics) dampens the EC sprouting/invasion. * indicates the statistical significance from the control. One-way ANOVA with Tukey *post hoc* tests was used for statistical analysis for both (**A**,**B**).

MicroRNA-150 is downregulated in patients with obesity [138], T1D [139,140], and T2D [121]. In high-fat-diet (HFD)-induced T2D mice, miR-150 is decreased in the plasma and retina [141,142]. Downregulation of miR-150 is also observed in the heart of STZ-diabetic rats [143] and in the ischemic retina of mice [144]. Inhibition of miR-150 promotes apoptosis [145], while its overexpression alleviates the apoptosis of cells under hypoxia [146], in which local hypoxia occurs in the diabetic retina [147,148]. Overexpression of miR-150 also protects the retinal vasculature from degeneration induced by oxygen-induced retinopathy, a model for hypoxia-induced angiogenesis [149]. Moreover, miR-150 is an intrinsic suppressor of inflammation [113]. Overexpression of miR-150 downregulates TNF-α and NFĸB induced by lipopolysaccharide (LPS) in endothelial cells [150]. Deletion of miR-150 (miR-150^−/−^) exacerbates the increase of IL-1β, IL-6, and TNF-α in mice with HFD-induced T2D [113]. We observed that plasma and retinal miR-150 is decreased in mice fed with an HFD even before diabetes develops [142]. The miR-150 knockout (miR-150^−/−^) mice with HFD-induced T2D display more severe retinal neural dysfunction and vascular pathologies compared with wild-type (WT) mice with HFD-T2D (Figure 1A) [141,142]. Therefore, decreased miR-150 correlates with the development of diabetes and may facilitate the development of DR by promoting apoptosis and inflammation in the neural and vascular retina.

### 4.2. The Targets of miR-150 in Diabetes

MicroRNAs often have many targets, and a single mRNA can also be targeted by multiple miRs [102,103,151], and the biological processes mediated by miRs and their targets are often tissue- and cell-type-specific [103,152]. Decreased miR-150 may promote apoptosis and inflammation in the diabetic retina through upregulating its downstream target genes. There are confirmed target genes of miR-150 that can regulate inflammation. In HFD-induced T2D mice, decreased miR-150 upregulates its target genes MYB proto-oncogene (*Myb*), ETS-domain transcription factor 1 (*Elk1*), and eukaryotic translation termination factor 1 (*Etf1*). Knocking down *Myb*, *Elk1*, or *Etf1* suppresses the inflammatory response by inhibiting the activation of B cells [113]. Early growth response 1 (*Egr1*) is another target gene of miR-150 [153], and knockdown of this target alleviates the diabetes-induced inflammation in mouse mesangial cells by downregulating pro-inflammatory factors (IL-1β, IL-6, and TNF-α) [154,155]. Moreover, these target genes of miR-150 (*Myb*, *Elk1*, *Etf1*, and *Egr1*) are also involved in the regulation of apoptosis. Knocking out *Myb* upregulates the apoptosis of mouse colorectal carcinoma cells [156], and overexpressing *Myb* decreases the production of ROS and alleviates the apoptosis in cardiomyocytes after hypoxia/reoxygenation injury [157]. Overexpression of ELK1 protein has been found to induce apoptosis in neurons by interacting with the mitochondrial permeability transition pore complex (PTP) [158]. Transfection of *Elk1* in the dendrites of primary neurons induces apoptosis [159], while inhibition of *Elk1* alleviates the apoptosis of neurons under oxygen–glucose deprivation [160]. Upregulated *Etf1* is associated with decreased apoptosis in mouse pre-osteoblast cells [161]. Increased expression of *Egr1* is associated with the apoptosis of squamous cell carcinoma cells and breast cancer cells, while knocking down *Egr1* mitigates apoptosis [162,163]. In addition to *Myb* and *Egr2* (direct targets of miR-150) that are known to promote angiogenesis by increasing the population of hemogenic endothelial cells [164] or upregulating vascular endothelial growth factor (VEGF) and its receptor 2 (VEGFR2) expressions [165,166], respectively, both VEGF and VEGFR2 are downstream of miR-150 [141,149]. VEGF and its principal receptor for angiogenesis VEGFR2 are both upregulated in diabetic eyes [167,168,169], and anti-VEGF therapies have been used to treat neovascularization in DR [169,170,171]. As miR-150 is downregulated in diabetes and DR, its downstream targets are upregulated and correlate with diabetes-associated inflammation, oxidative stress, apoptosis, and pathological angiogenesis. Thus, miR-150 is not only a biomarker for diabetes and DR, but it could also be a potential therapeutic target for treating diabetes-associated chronic diseases and DR.

### 4.3. miR-150 and Inflammation in the Diabetic Retina

As mentioned previously, inflammation is a major contributor to DR [72,172]. Chronic meta-inflammation is a hallmark of obesity and obesity-associated type 2 diabetes (T2D) [173,174], but numerous studies have indicated that intraocular rather than systemic inflammation is more closely associated with the vascular complications in DR [46,74,175,176,177,178,179,180,181,182,183,184]. Interestingly, diabetic patients who also have retinitis pigmentosa (RP), a congenital blindness with initial degeneration of rod photoreceptors, rarely develop DR [185,186,187], and there is a clear inverse correlation between RP and DR [185,186]. The RP patients who had been diabetic for nearly 40 years developed other non-retinal vascular complications, but none had retinal microaneurysms, exudates, or any clinical DR [185,186,187]. In mice, genetic deletion of rod photoreceptors or pharmacological inhibition of photoreceptors reduces retinal inflammation and alleviates progression of DR [188,189]. Therefore, retinal photoreceptors are a major source of intraocular inflammation and directly contribute to vascular abnormalities in diabetes [6,22,23,24,33,34,36].

MiR-150 is an intrinsic suppressor of inflammation [114] since it suppresses the expression of pro-inflammatory molecules and cytokines, including nuclear factor kappa B (NF-ĸB), TNFα, IL1β, and IL6 [113,190,191,192,193,194]. Overexpression of miR-150 downregulates lipopolysaccharide (LPS)-induced expression of TNF-α and NF-ĸB in endothelial cells [150], while deletion of miR-150 in mice (miR-150^−/−^) augments LPS-stimulated inflammatory responses [113]. MiR-150^−/−^ mice with HFD-induced T2D have further elevated serum pro-inflammatory cytokines (TNFα, IL1β, IL6, CCL2) and lower anti-inflammatory cytokine (IL10) [113]. Compared with wild-type (WT) mice with HFD-induced T2D, these miR-150^−/−^-T2D mice display more severe T2D with increased glucose intolerance and insulin resistance [113] and significantly reduced retinal light responses [141,142]. Thus, miR-150 exhibits anti-inflammatory [113] properties, and diabetes-associated decrease of miR-150 may contribute to ocular inflammation and further exacerbate the development of DR.

We previously showed that miR-150^−/−^-T2D mice have more severe inflammation in photoreceptors and exacerbated vascular degeneration compared with the WT-HFD mice [142]. Since the biological processes mediated by microRNAs and their targets are often tissue- and cell-type-dependent as stated earlier [152,195], after screening the top 30 predicted target genes of miR-150 [113,193] and identifying new bona fide targets that are pro-inflammatory [113], multiple transcription factors including the eukaryotic translation termination factor 1 (*Etf1*), early growth response 1 (*Egr1*), MYB proto-oncogene (*Myb*), and *ETS-domain transcription factor 1 (Elk1)* were found to be expressed in retinal photoreceptors and endothelial cells [196]. Downregulation of miR-150 correlates with an upregulation of *Etf1*, *Egr1*, *Myb*, and *Elk1* and pro-inflammatory cytokines, while overexpression of miR-150 or knocking down any of these transfection factors decrease the expression of pro-inflammatory cytokines in cultured adipose B lymphocytes [113].

In the diabetic retina, photoreceptors are one of the major sources of retinal inflammation [175,189]. Using cultured murine photoreceptors treated with palmitic acid (PA) to mimic obesity-associated T2D, we found that PA elicited an increase of phosphorylated NF-ĸB (pP65), an inflammation marker, which persisted for 24 h and correlated with a persisting decrease of miR-150 and increase of *Elk1*. However, PA elicited only temporary increases of *Etf1*, *Egr1*, or *Myb*, although these three transcription factors are direct downstream targets of miR-150 and expressed in the neural retina [196]. Overexpression of miR-150 or knocking down *Elk1* not only decreased the expression of ELK1 (protein) but also relieved PA-induced increase of inflammation.

Phosphorylated ELK1 at S383 (pELK1_S383_) translocates from the cytoplasm to the nucleus at which time it then activates its downstream genes to promote inflammation [197,198]. The level of pELK1_S383_ was increased in the retinal outer nuclear layer of obesity-associated T2D mice and the nuclei of palmitic acid-treated murine photoreceptors in cultures, and the increased nuclear pELK1_S383_ correlated with the upregulated pP65. Deletion of miR-150 not only upregulated ELK1 but also cytoplasmic pELK1_S383_ in photoreceptors. The miR-150^−/−^ mice with obesity-associated T2DR had further exacerbated retinal photoreceptor inflammation compared with the WT-T2DR mice, and the photoreceptor inflammation correlated with an increase of pELK1_S383_ in the retinal outer nuclear layer [196]. The nuclear/cytoplasmic (N/C) ratio represents the cytoplasm-to-nucleus translocation of pELK1_S383_, and PA treatments increased the N/C ratio of pELK1_S383_ in cultured photoreceptors, and knocking down *Elk1* decreased nuclear pELK1_S383_ and the N/C ratio of pELK1_S383_ in PA-treated photoreceptors, which also correlated with the downregulation of pP65. Hence, T2D-associated inflammation in photoreceptors was in part mediated by a decrease of miR-150 that caused an increase of nuclear pELK1_S383_ and led to photoreceptor inflammation. Therefore, overexpression of miR-150 or knocking down of *Elk1* may restrain the development of DR by mitigating the inflammation in the neural retina, especially in photoreceptors [196].

### 4.4. miR-150 and Neural Apoptosis in the Diabetic Retina

The neural retina has the highest oxygen consumption rate among all tissues, including the brain [199], thus making it (especially the photoreceptors) prone to hypoxia-induced apoptosis. There is local hypoxia in the diabetic retina, and among retinal neurons, photoreceptors undergo apoptosis shortly after the onset of diabetes [38,39]. Patients with T1D have thinner neural layers in the retina and visual dysfunction before the diagnostics of DR [23]. Adolescents with T2D for an average of two years have reduced retinal thickness and dampened light responses [27]. The loss of retinal neurons starts 10 weeks after STZ-induced T1D in mice [33], and neuronal apoptosis in the retina occurs in T2D (db/db) mice from 20 weeks of age [35]. Apoptotic photoreceptors can be detected in STZ-diabetic rats 4 weeks after the onset of diabetes [38]. In addition, the dysfunction of photoreceptors in STZ-diabetic mice is associated with the reduced thickness of the outer nuclear layer [200]. Furthermore, diabetic patients with retinitis pigmentosa (RP), a genetic disease with loss of photoreceptors, rarely develop DR, even though these patients develop other diabetes-related vascular diseases [44,45]. In a mouse model of RP, during the period when photoreceptors are undergoing apoptosis, the retinal vasculature is also degenerating. Once all photoreceptors have died, the vascular degeneration stops [46]. Hence, photoreceptor apoptosis in diabetes not only contributes to the neural dysfunction but may also adversely impact diabetic microvascular complications [46,200] and lead to DR.

Subsets of miRs are known to regulate cell proliferation and apoptosis [139]. Among them, inhibition of miR-150 promotes apoptosis of cells under hypoxia [145], and local hypoxia occurs in the early diabetic retina [147,148]. Overexpression of miR-150 alleviates the apoptosis of hypoxic cells [146]. In our HFD/obesity-associated T2D mouse model, not only did HFD-T2D mice have more severe retinal neural dysfunction and apoptotic photoreceptors versus mice fed with a normal diet [141,142], the miR-150^−/−^ mice with HFD-T2D had even more neural dysfunction and the highest numbers of apoptotic photoreceptors compared with the WT mice with HFD-T2D [196]. To further verify the relationship between miR-150 and photoreceptor apoptosis, we treated cultured photoreceptors with PA to elicit cell apoptosis. Interestingly, knocking down miR-150 in photoreceptors caused a significant increase in apoptosis regardless of PA treatments. However, transfections with miR-150 mimics did attenuate the PA-induced apoptosis. Thus, overexpression of miR-150 only in photoreceptors alone might not be enough to overturn PA-induced apoptosis, but an adequate level of miR-150 is necessary for the survival of photoreceptors [196].

Among the major targets of miR-150 expressed in photoreceptors, *Elk1* is known to promote apoptosis in neurons [159,201], as overexpression of ELK1 protein has been found to promote apoptosis in neurons. Transfection of *Elk1* in the dendrites of primary neurons induces apoptosis [159], while inhibition of *Elk1* alleviates the apoptosis of neurons under oxygen–glucose deprivation [160]. As mentioned previously, the activation of ELK1 requires its phosphorylation, and phosphorylation of ELK1 at threonine 417 (pELK1_T417_) specifically is essential for ELK1-mediated neuronal apoptosis [158]. Thus, we analyzed the levels of ELK1 and pELK1_T417_ in the inner and outer segments of photoreceptors (IS + OS) as well as in the outer nuclear layer (ONL) from the retinas of HFD-T2D mice [196]. We found that the levels of ELK1 in the cytoplasm (IS + OS) and nuclei (ONL) of photoreceptors were significantly increased in both WT and miR-150^−/−^ mice with HFD-T2D. Knockout of miR-150 (miR-150^−/−^) upregulated pELK1_T417_ in the IS + OS of photoreceptors, while increased pELK1_T417_ was observed in the ONL of all HFD-T2D retinas. It is possible that HFD-induced apoptosis in photoreceptors was mediated by an increase in nuclear pELK1_T417_, and the upregulation of cytoplasmic pELK1_T417_ caused by miR-150 knockout exacerbated the HFD-induced apoptosis.

To further determine the relationship of miR-150, ELK1/pELK1_T417_ and photoreceptor apoptosis, we employed PA-induced apoptosis in cultured photoreceptors [196]. After photoreceptors were treated with PA, the levels of ELK1 significantly increased in a time-dependent manner, which correlated with increased apoptosis. As treatments with PA (24 h) significantly increased ELK1 in all cells, knocking down miR-150 further elevated the PA-elicited increase in ELK1. However, overexpression of miR-150 did not attenuate the PA-induced increase of ELK1 suggesting that overexpression of miR-150 is not sufficient to downregulate PA-stimulated ELK1, which echoes that overexpression of miR-150 in photoreceptors alone might not be enough to overturn PA-induced apoptosis.

In order to verify the functions of ELK1 and pELK1_T417_ in regulating apoptosis in photoreceptors, we knocked down *Elk1* with siRNA (si*Elk1*) in cultured photoreceptors and found that PA-elicited increase of ELK1 was blocked by si*Elk1*, and knocking down *Elk1* decreased cytoplasmic pELK1_T417_ and also arrested the PA-induced increase in nuclear pELK1_T417_. Thus, knocking down *Elk1* effectively inhibited the PA-elicited increase in ELK1 and pELK1_T417_. Unfortunately, PA-induced apoptosis was not dampened by si*Elk1*, so knockdown of *Elk1* alone cannot attenuate PA-induced apoptosis, which is consistent with our data where upregulation of miR-150 in photoreceptors alone was not enough to conquer PA-induced apoptosis [196].

Cell apoptosis can be mediated by the mitochondrial permeability transition pore complex (PTP) that initiates mitochondrial swelling and membrane potential depolarization that leads to cell death [202]. There is a protein–protein interaction between ELK1 and PTP in the brain, and cytoplasmic ELK1 can be isolated from purified mitochondrial fractions. Furthermore, cell apoptosis induced by *Elk1* overexpression can be blocked by a PTP inhibitor in cultured primary neurons [201]. Thus, under T2D conditions, upregulated cytoplasmic pELK1_T417_ would have increased interactions with mitochondrial PTP, which might further accelerate photoreceptor apoptosis. However, while overexpression of miR-150 or downregulation of *Elk1* decreases cytoplasmic pELK1_T417_, it does not reduce nuclear pELK1_T417_ or overcome PA-elicited apoptosis. In PA-treated photoreceptors, the nuclear/cytoplasmic (N/C) ratio of pELK1_T417_ remains comparable with cells with/without overexpression of miR-150 and cells with/without knockdown of *Elk1*. The N/C ratio represents the cytoplasm-to-nucleus translocation of pELK1_T417_, which is important for trans-activating the downstream targets of *Elk1* and regulating apoptosis [203,204]. The translocation of pELK1_T417_ to the cell nucleus correlates with increased apoptosis in neurons [205]. Therefore, in addition to dampening the expression of ELK1, blocking the translocation of pELK1_T417_ to the nucleus may be more critical to mitigate diabetes-associated apoptosis in photoreceptors [196].

### 4.5. miR-150 and Angiogenesis

As neural miR-150 is important for protecting the retina under diabetic insults (described above), decreased miR-150 in vascular endothelial cells may also contribute to ocular angiogenesis. Overexpression of miR-150 in mouse eyes protects the retinal microvasculature from degeneration induced by oxygen-induced retinopathy, a model for pathological angiogenesis in retinopathy of prematurity [205,206], and deletion of miR-150 exacerbates T2D-associated microvascular leakage and degeneration [141,142]. Since well-known diabetic mouse models do not have pathological neovascularization like in PDR patients, we used a three-dimensional collagen matrix culture system of endothelial cells to evaluate the effect of endothelial miR-150 in vascular sprouting, an indicator of neovascularization (Figure 1). We found that overexpression of miR-150 dampened endothelial sprouting and invasion, but inhibition of miR-150 did not affect normal endothelial sprouting (Figure 1B). Furthermore, overexpression of miR-150 decreased the expression of VEGFR2 in cultured endothelial cells [141]. Interestingly, VEGFR2 is not a direct downstream target of miR-150, since the VEGFR2 gene lacks compatible paired sequences. Conversely, the miRNAs predicted to target the 3′-UTR (1479 bp) of the mouse VEGFR2 gene do not include miR-150 [141]. One direct target of miR-150 that may regulate the expression of VEGFR2 is the transcription factor *Myb*, which binds to a 5′-YAACKG-3′ sequence in the promoter region and regulates the expression of a group of genes involved in cell lineage- and fate-determination in the immune system. The gene encoding VEGFR2 (*Vegfr2*) has four *Myb* binding sites in its promoter region, so *Vegfr2* can be turned on by *Myb* [67]. Overexpression of *Myb* increases the population of hemogenic endothelial cells during embryonic development [68]. We found that overexpression of miR-150 also decreased the expression of MYB in cultured endothelial cells [141], making *Myb* the most likely downstream target of miR-150 that regulates VEGFR2 expression in vascular endothelial cells. Table 2 is a list of the direct targets of miR-150 discussed above.

## 5. Conclusions

**Figure 2:**
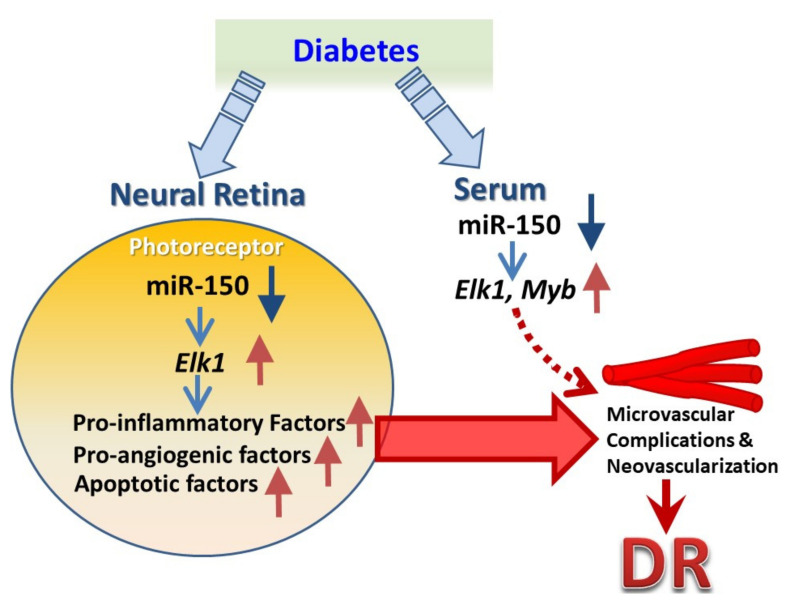
An illustration of how miR-150 contributes to microvascular complications and neovascularization in diabetic retinopathy (DR) as summarized in the conclusion.

As decreased miR-150 in the diabetic retina correlates with the development of DR, the action and downstream targets of miR-150 in neural versus vascular retina are different, but all contribute to the pathogenesis of DR (Figure 2). In the neural retina, diabetes-associated decrease of miR-150 promotes inflammation and apoptosis of photoreceptors via *Elk1*, which contribute to the microvascular degeneration in DR. In the retina vasculature, diabetes-associated decrease of miR-150 promotes endothelial cell sprouting via *Myb*, which contributes to neovascularization in DR (Figure 2). MiR-150 is expressed in the neural and vascular retina and is also abundant in the circulation, so diabetes-elicited changes in circulating miR-150 are reported in both T1D and T2D patients. Hence, miR-150 is not only a biomarker for DR, it is indeed involved in the pathogenesis and the disease progression of DR. With extensive investigation of miR-150 as an example, diabetes-associated changes in other miRs not only serve as biomarkers to indicate the pathological progression of DR, but they might also actively contribute to the pathogenesis of DR.

## Figures and Tables

**Table 1:** A list of microRNAs changed in diabetes reviewed in Section C.

	Upregulated miR	Downregulated miR
In T1D patients	miR-29a, miR-30b, miR-148a, miR-181a, and miR-200a [119,121]	miR-21a, miR-93, miR-126, miR-146a, and miR-150 [119,120,121]
In STZ-induced T1D rodents	miR-195 [130,131], miR-495 [125].	
	In STZ-induced diabetic rats, at least 86 miRs are significantly altered in the retina [115,117,118]	
In T2D patients and T2D mice		miR-15a, miR-20b [137], miR-21 [127], miR-24, miR-93, miR-126, miR-146a, miR-150, miR-191, miR-197, miR-320, and miR-486 [122,123]

**Table 2:** A list of the direct targets of miR-150 discussed in Section D.

Direct Target of miR-150	Reference
*Cxcr4* (C-X-C chemokine receptor type 4)	Liu (2015) [149]
*Dll4* (Delta like ligand 4)	Liu (2015) [149]
*Egr1* (Early growth response 1)	Ying (2016) [113] Shen (2019) [153]
*Egr2* (Early growth response 2): verified downstream angiogenic targets are VEGF and VEGFR2	Nagarajan (2001) [165] Joseph (1988) [166]
*Elk1* (ETS-domain transcription factor)	Shi (2016) [141] Zhu (2017) [145] Ying (2016) [113] Yu (2020, 2021) [196,207]
*Etf1* (Eukaryotic translation termination factor 1)	Ying (2016) [113]
*Fzd4* (Frizzled-4)	Liu 2015 [149]
GPNMB (Glycoprotein nonmetastatic melanoma protein B): verified downstream angiogenic target is Neuropilin-1 (NRP-1)	Maric (2013) [208] Becker (2005) [209] Narasaraju (2015) [210]
*Myb* (MYB proto-oncogene): verified downstream angiogenic target is VEGFR2	Ishida (2012) [211] Dai (2006) [164]

## Data Availability

Not applicable.
